# A common garden experiment in the wild reveals heritable differences in migration tendencies among brown trout populations

**DOI:** 10.1111/jfb.16068

**Published:** 2025-02-06

**Authors:** Thomas E. Reed, Robert Wynne, Jamie Coughlan, Patrick Gargan, Joshka Kaufmann, Karl. P. Phillips, Adrian Rinaldo, Russell Poole, Philip McGinnity

**Affiliations:** ^1^ School of Biological, Earth and Environmental Sciences University College Cork Cork Ireland; ^2^ Environmental Research Institute University College Cork Cork Ireland; ^3^ BioGradTM Liverpool UK; ^4^ School of Biological and Environmental Sciences Liverpool John Moores University Liverpool UK; ^5^ Inland Fisheries Ireland Dublin Ireland; ^6^ Marine Institute Newport Ireland; ^7^ Canadian Rivers Institute University of New Brunswick Fredericton New Brunswick Canada

**Keywords:** facultative, fitness, microsatellite, migratory, partial migration, smolt, tactic

## Abstract

We undertook a common garden experiment in the Burrishoole catchment, western Ireland, to test for heritable life‐history differences among neighboring brown trout (*Salmo trutta* L.) populations that exhibit neutral genetic divergence. Experimental crosses were made using either local females (obtained from a below‐waterfalls section of the Rough River within the Burrishoole) or females from the Erriff River—a neighboring catchment that currently produces a stronger run of anadromous migrants than the Burrishoole. Each female was mated to three different types of males: Rough Below‐Falls, Rough Above‐Falls (resident males obtained from above the waterfalls), and Erriff. Offspring from the resulting six crosses were introduced as unfed fry into a stretch of the Rough River bounded upstream by the waterfalls and downstream by a Wolf‐type fish trap (Rough River Downstream Trap, RRDT). Genetic parentage analysis (16 microsatellite markers) was then used to assign offspring sampled at various time points and locations back to cross type. No differences in parr survival rates (electrofishing in the Rough River) were found among the crosses, but parr moving downstream (intercepted at the RRDT) were skewed toward the Erriff female × Erriff male cross, with a deficit assigning to the Rough Below‐Falls female × Rough Above‐Falls male cross. Smolts leaving fresh water (sampled at two sea‐entry traps) were assigned disproportionately to crosses involving one or two Erriff parents. Offspring from pure Burrishoole crosses were more likely to become putative spawners than those from crosses involving one or two Erriff parents, pointing toward possible local adaptation. These results are consistent with heritable variation in migratory tendencies—a key aspect of intraspecific biodiversity that warrants protection—and with previous suggestions that the Burrishoole system may have evolved recently toward reduced anadromy following a novel and catastrophic anthropogenic change.

## 1 INTRODUCTION

Migratory patterns vary greatly both across and within species, encompassing a range of physiological, behavioral, and life‐history traits (Dingle & Drake, 2007). Understanding the extent to which variation in migratory traits is determined by genetic versus environmental effects is a difficult but important challenge (Charmantier & Gienapp, 2014; Liedvogel et al., 2011; Pulido, 2011), with implications for conservation biology and management of harvestable wild stocks. If migratory traits are heritable, this can allow for evolutionary response to environmental change (Kane et al., 2022; Phillis et al., 2016; Pulido & Berthold, 2010; Theriault et al., 2008), while adaptive genetic divergence among populations can foster increased resilience of metapopulations to anthropogenic stressors (Carlson & Satterthwaite, 2011; Schindler et al., 2010).

Common garden experiments (CGEs) are a classical approach for testing whether phenotypic divergence among populations has a genetic basis (de Villemereuil et al., 2016). If phenotypic differences persist among individuals from different population backgrounds when reared under the same environmental conditions, this points toward heritable population differences. Oviparous fishes are particularly well suited to CGEs because broodstock can be relatively easily collected from wild environments, kept in captivity, and then stripped of their gametes to create experimental families. The progeny can then be introduced into a “common garden,” which could involve a controlled laboratory environment (e.g., Archer et al., 2019, 2020; Doctor et al., 2014; Harvey et al., 2016), semi‐natural streams (e.g., Robertsen et al., 2019; Solberg et al., 2020), or a fully natural wild environment (e.g., McGinnity et al., 1997, 2004, 2003; O'Toole et al., 2015; Skaala et al., 2012, 2019).

Salmonid fishes are popular models for such work, given their pronounced phenotypic diversity and highly structured populations. A spectrum of migration tactics occurs, including anadromy (migration to saltwater for a period of growth followed by a return spawning migration to fresh water), potamodromy (migrations within fresh water, e.g., from small streams to larger rivers, or from streams/rivers to lakes), and residency (Dodson et al., 2013; Klemetsen et al., 2003; Quinn & Myers, 2004). Many salmonid populations also exhibit facultative/partial migration (Chapman et al., 2012), wherein individuals are capable of adopting either a resident or a migratory tactic, with the ratio of residents to migrants varying among populations and between the sexes within populations (Dodson et al., 2013; Ferguson et al., 2019; Kendall et al., 2014; Nevoux et al., 2019; Sloat et al., 2014). Although often described in simple binary terms, facultative migration can involve considerable complexity, with many life‐history trajectories possible (Birnie‐Gauvin et al., 2019).

The environmentally cued threshold model (Tomkins & Hazel, 2007) has been particularly useful to conceptualize how alternative migratory tactics can emerge from an interplay between a genetically variable threshold and an environmentally sensitive status trait (“liability”) such as energetic status or physiological condition (Buoro et al., 2012; Buzatto et al., 2015). Whether migration or residency is adopted depends on whether the liability exceeds the threshold during a sensitive period in early ontogeny. Thus, identical genotypes can express different migratory tactics if they experience different environmental conditions, via phenotypic plasticity. Conversely, if the genetic threshold differs among individuals or populations, variation in migration tactics results even if all individuals experience the same environment (Debes et al., 2020; Lepais et al., 2017; Piché et al., 2008). Additional aspects of migratory syndromes, such as the age, seasonal timing, and destination of migration, may be further shaped by some combination of genetic and environmental influences (Birnie‐Gauvin et al., 2021; Ferguson et al., 2019; Nevoux et al., 2019).

The brown trout (*Salmo trutta*) is among the most phenotypically diverse vertebrates on Earth (Ferguson & Prodöhl, 2022; Klemetsen, 2013; Lobón‐Cerviá, 2017), in which a broad range of migratory strategies are found (Cucherousset et al., 2005; Ferguson et al., 2017, 2019; Jonsson, 1985; Nevoux et al., 2019). Spawning occurs in streams, with the juveniles called fry during early life as they transition to exogenous feeding and establish territories, and parr toward the end of their first summer when they develop distinctive dark blotches (parr marks) along their flanks. Parr gradually drop downstream as they grow, in search of deeper/faster water, but those exhibiting limited movements within their natal streams are known as residents. Individuals adopting an active downstream migration tactic to more productive habitats are then known as smolts, with some authors reserving the term exclusively for anadromous marine migrants, but others using it also for potamodromous migrants that migrate to larger rivers or lakes (given similar morphological, physiological, and behavioral changes occur in both cases; Ferguson et al., 2019). Although various lines of evidence suggest that the propensity for migration is heritable in this species and other salmonids (reviewed by Ferguson et al., 2017, 2019), there remains a paucity of direct evidence for genetic divergence among populations in migration‐related traits. Previous common garden or reciprocal transplant experiments have been undertaken with brown trout, but these have been limited to artificial (Archer et al., 2019, 2020) or semi‐artificial (Vainikka et al., 2023) settings, or else just two populations (Jonsson, 1982; Olsson et al., 2006). Here, we report on the results of a single generation, multiyear, CGE in the wild involving three different *S. trutta* populations: two from the Burrishoole catchment, western Ireland, and one from the Erriff catchment, located ~30 km to the south (straight‐line distance between river mouths). The Burrishoole system formerly had a strong run of anadromous upstream migrants, but a collapse occurred in 1989/1990 (de Eyto et al., 2016; Poole et al., 1996, 2007). Poole et al. (2007) speculated that a sudden drop in marine survival and growth around this time, associated with increased salmon farming activity in the region, may have driven rapid evolution of reduced anadromy, which would require the trait to be heritable. Although life‐history changes have also occurred within the Erriff system, with salmon farming again implicated, the adult sea trout post‐spawner (“kelt”) numbers had recovered by the late 1990s and early 2000s following a collapse in 1989/90, and the annual downstream smolt run (i.e., outmigrant juveniles) remained stable between 1986 and 2004 (Gargan et al., 2016). In contrast, the adult sea trout population has shown little to no recovery in the Burrishoole system, while smolt numbers have also declined considerably, despite fry/parr numbers in the streams remaining apparently unchanged (Marine Institute data, 2023). We thus hypothesized that the current genetic propensity for anadromy is lower in the Burrishoole than in the Erriff. To test this, we created experimental crosses involving pure Burrishoole families (both parents obtained from a below‐waterfalls section of the Rough River, a major tributary in the Burrishoole system), pure Erriff families, and reciprocal F1 hybrids. As a control group, we also crossed Rough Below‐Falls (RB) females and Erriff (ER) females against males obtained from an above‐falls section of the Rough River (RA) that harbors a resident population of brown trout that presumably experiences selection against migration. The offspring of these six experimental crosses were released as fry into a below‐falls section of the Rough River and their survival, growth, and movements subsequently monitored using molecular parentage assignment to determine fish identity.

Assuming the RB population to have a somewhat higher intrinsic propensity for anadromy compared to the RA population, but not as high as the ER population, we expected anadromy rates in the offspring (production of downstream migrating smolts) to exhibit the following pattern with respect to cross type: ER × ER > ER × RB/RB × ER > ER × RA ≈ RB × RB > RB × RA, assuming an additive genetic basis to anadromy and no parent‐of‐origin effects (Guilmatre & Sharp, 2012) in the hybrids. Moreover, if local adaptation is important at the catchment level, then offspring with two Burrishoole parents (who are in their “home” environment) should perform better, in terms of parr growth rates and survival through to spawning, than offspring with two Erriff parents (which are in an “away” environment), with hybrids exhibiting potentially intermediate performance.

## 2 METHODS

### 2.1 Study areas

**FIGURE 1:**
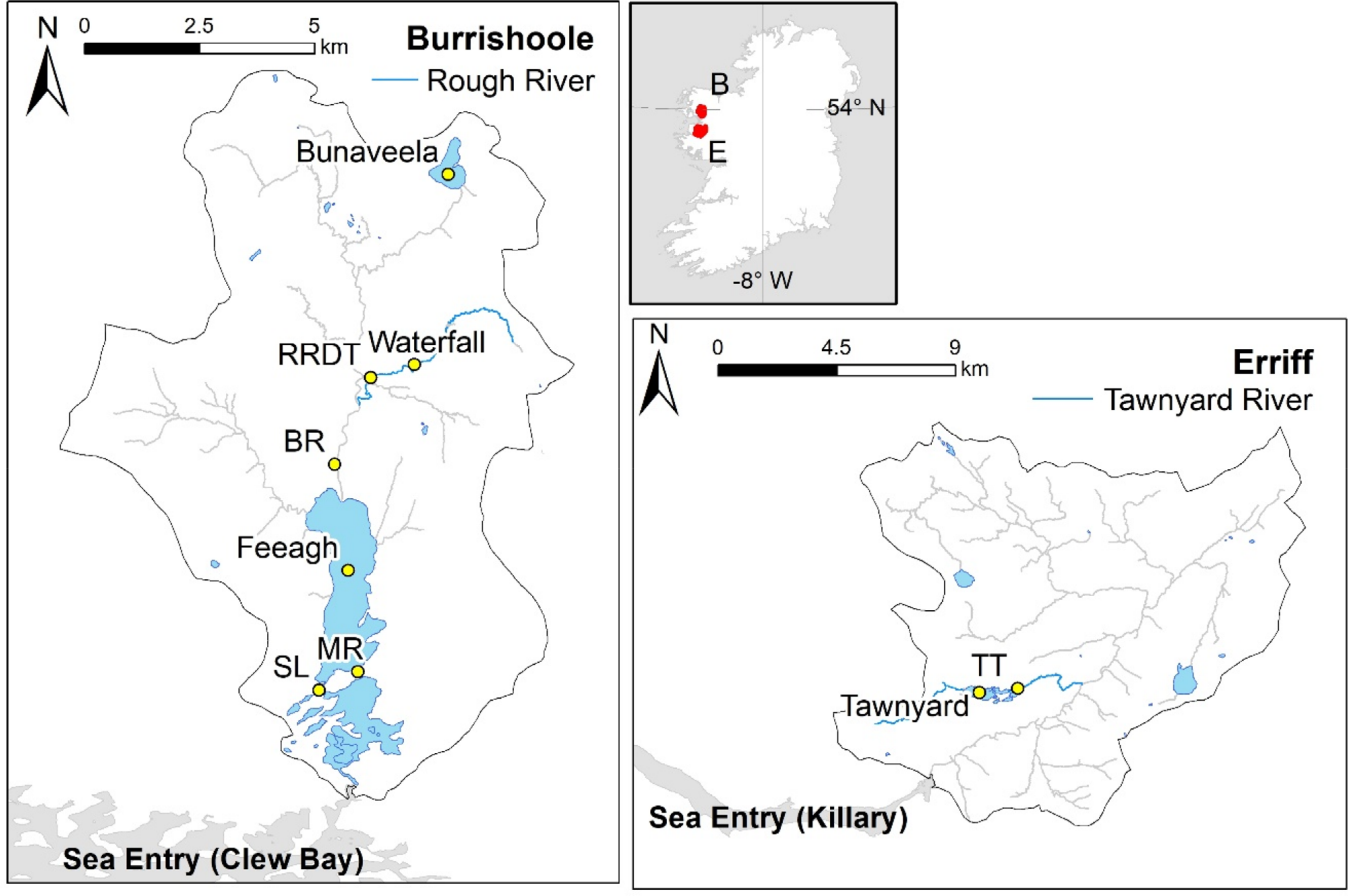
Map of the Burrishoole and Erriff catchments, western Ireland. The common garden experiment was conducted in the Rough River (highlighted in blue) within the Burrishoole catchment, in a section bounded upstream by the waterfall and downstream by the Rough River Downstream Trap (RRDT). The Rough River connects to Lough Feeagh via the Black River (BR). Trapping facilities at the outlet of Lough Feeagh (MR, Mill Race; SL, Salmon Leap) allowed for monitoring of smolts leaving fresh water. Broodstock for the experiment were obtained from above and below the waterfall in the Rough River (Burrishoole catchment) and from various sites throughout the Erriff catchment. Sea trout smolts and kelts are monitored at the Tawnyard Trap (TT) in the Erriff system, just downstream of Lough Tawnyard.

The Burrishoole system (53.93° N 09.57° W; Figure 1) consists of over 45 km of shallow, oligotrophic and poorly buffered streams and three main lakes (Lough Bunaveela, fresh water, 46 ha; Lough Feeagh, fresh water, 410 ha; and Lough Furnace, brackish, 172 ha), emptying into Clew Bay (de Eyto et al., 2020). Strongly influenced by the Atlantic Ocean (Jennings et al., 2000), the climate is temperate and oceanic, with mild winters and cool summers. Brown trout in the system can be stream residents, potamodromous (migration to larger rivers or lakes), semi‐anadromous (migration to brackish areas), or anadromous (migration to full salt water). Between 1971 and 1979, a mean of 2503 silvered trout returned to the catchment; this fell remarkably after the aforementioned collapse to a mean of 75 silvered returns between 2000 and 2009 (Poole et al., 2007). The numbers have remained low since, with a mean of 49 silvered returns between 2020 and 2024 (Marine Institute, unpublished data). From 1971 to 1979, a mean of 7465 smolts and autumn parr were enumerated on their downstream migration; downstream migrant numbers were maintained for a few years after the sea trout collapse in 1989/90 but had fallen to a mean of 1572 by 2000–2009 (Poole et al., 2007) and a mean of 971 by 2020–2024 (Marine Institute, unpublished data).

The Erriff River in the west of Ireland (53.62° N 09.67° W; Figure 1) is approximately 30 km long, and the catchment comprises mostly blanket peat habitat. The Black River (3 km long and approximately 8 km upstream from the tidal limit) is the main tributary; it leads to Tawnyard Lough (56 ha) and is the principal sea trout fishery. A downstream trapping facility has been in operation just downstream of Tawnyard Lough since 1985. The numbers of sea trout kelts monitored at the Tawnyard trap averaged at 506 between 1985 and 1988, dipped to 62 in 1990 and 116 in 1991, and then averaged at 533 between 1992 and 2004 (Gargan et al., 2016). The numbers of smolts enumerated did not change (no linear temporal trend) between 1986 and 2004, averaging at 2209 (Gargan et al., 2016).

### 2.2 Experimental design

Broodstock for the CGE were collected in the 2016/2017 spawning season (winter) from three locations: (a) below an impassable waterfall in the Rough River (also known as the Srahrevagh River), a key tributary in the Burrishoole system known to produce a mix of migratory and river‐resident trout; (b) above the waterfall in the Rough, where the trout are entirely river‐resident and where selection presumably disfavors migration; and (c) from the Erriff catchment (various sites throughout the system capable of receiving anadromous migrants). The migration history of the broodstock was not known, but many of the Erriff fish showed signs (silvering, large body size) of having been to sea. The three populations showed evidence for neutral genetic divergence (see supplementary materials; pair‐wise Weir & Cockerham F_ST_ based on 16 microsatellite markers: Rough Below vs. Erriff = 0.102; Rough Above vs. Erriff = 0.157; Rough Below vs. Rough Above = 0.049).

Of the nine possible crosses between these three populations, only six were feasible owing to the very low fecundity of resident females from the above‐falls Rough River population. Therefore, only RB females and ER females (F) were used, and each was crossed against males (M) from the three populations (RB, ER, RA), yielding six crosses or genetic groups: 1 = RBF × RBM; 2 = RBF × RAM; 3 = RBF × ERM; 4 = ERF × RBM; 5 = ERF × RAM; 6 = ERF × ERM. The fertilized eggs were incubated at an experimental hatchery facility in the Burrishoole catchment located just to the west of the Mill Race sea‐entry trap (Figure 1), supplied with water piped directly from Lough Feeagh. Full details on the broodstock, crossing procedures, and hatchery rearing are given in the supplementary materials.

Exact counts of the number of fertilized eggs per full sibling family were available, but for logistical reasons, it was not possible to incubate eggs from each full‐sib family separately until the unfed‐fry stage (the point at which the alevins have almost completely used up their yolk sacs and are nearly ready for transitioning to exogenous feeding). Thus, only estimates of the final number of unfed fry per full‐sib family were available, which were then aggregated to give corresponding estimates per cross type (assuming egg to unfed fry survival was random with respect to cross type; see supplementary materials). An estimated total of 12,833 unfed fry across the six cross types were introduced (i.e., stocked) into the upper third of the experimental stretch of the Rough River in March 2017, bounded upstream by a series of large waterfalls and downstream by a trap (the Rough River Downstream Trap, RRDT; Figure 1). Naturally spawned *S. trutta* (produced by non‐experimental parents) from a range of natal cohorts were also present in the river throughout the course of the experiment, as wild adults were not precluded from spawning in the Rough River.

### 2.3 Sampling the offspring

Monitoring of downstream movements out of the experimental stretch of river via the RRDT began immediately and continued daily for the duration of the experiment (from March 2017 to the end of December 2021). All trout captured were measured for fork length (mm) and weight (g), and tail fin clips were taken and preserved in 99% molecular‐grade ethanol for subsequent genetics work. Any fish above 65 mm was fitted with a passive integrated transponder (PIT) tag (RFID HDX 4005, Biomark). In addition to fish captures via the RRDT, the whole length of the experimental river was electrofished on 29 different days between March 2017 and September 2020 using backpack electrofishing (Hans Grassl GmbH, Germany), with sampling effort focused on the summer/early autumn months. Fish caught by electrofishing were processed as per fish caught in the RRDT.

To investigate trout movements over the duration of the experiment, a network of swim‐through, cross‐channel HDX PIT antennae readers was installed in strategically placed sites in the Rough River (above and below the trap). Anadromous migrants leaving fresh water were sampled at the Mill Race and Salmon Leap downstream sea‐entry traps (Figure 1), and tissue samples were taken for genetics. These sea‐entry traps were monitored daily during periods of low migration, and twice daily during periods of peak migration. Fixed antennas (Biomark IS1001 readers) were also in place at the inlet of both sea‐entry traps, and these were augmented by hand‐held scanners (HPR+ and HPRLite; Biomark) to identify any tagged fish intercepted in the traps that were missed by the antennae. All fish identified on either the fixed antennae or the hand‐held scanners were sampled.

### 2.4 Molecular work and parentage analysis

DNA extraction and amplification using PCR and genotyping protocols were as described in Wynne et al. (2023). Sixteen microsatellite loci and the SalmoY sex identity locus were amplified across two separate multiplex reactions (see supplementary materials). Mean allelic richness across the loci was 12.24, ranging from 2.67 to 28.65 (across *n* = 6860 juveniles sampled in the wild as part of a wider project). Parentage assignments were carried out using the programme FAP (Taggart, 2007), which estimates exclusion‐based family assignment probabilities within family mixtures where all parental genotypes are known. Samples with missing genotypes at ≥7 loci were excluded. The “allele size tolerance” parameter was set to zero, and the “allele mismatch tolerance” parameter was set to two, with the programme accounting and correcting for any mismatches resulting from scoring errors. We also ran a parentage analysis as a check, using the programme COLONY (Jones & Wang, 2010), and the assignments to cross type matched exactly the FAP assignments. Fish that were not assigned to any of the experimental families were assumed to have been produced by unsampled parents that spawned naturally in the wild. The concatenated multilocus genotype was used as a “genetic tag” to identify matching pairs of samples ostensibly from the same individual captured on two or more occasions (with only exact matches considered, i.e., not allowing for any mismatching loci, to be conservative). If the fish was PIT tagged and then subsequently recaptured, the PIT tag information was linked to any genetic tag information, allowing it to be identified as the same individual across multiple sampling occasions based on either or both.

### 2.5 Statistical analysis

χ^2^ tests, with *p*‐values computed by Monte Carlo simulation with 2000 bootstraps (*chisq.test* function in R), were used to test for differences between the proportions of fish in a given sample assigning to each experimental cross and the estimated proportions in the introduced fry (baseline). χ^2^ tests were similarly used to compare the proportions assigned to each cross type between pairs of samples of interest. In cases where the same individual (as identified based on either its multilocus genotype or its PIT tag, or both) was recaptured multiple times within the same sample, only the first capture was retained to avoid double counting of individuals. Exact binomial tests were used to test for sex ratio differences arising from parity.

General linear models (GLMs) were used to test for differences in parr length (electrofishing and RRDT data) among offspring assigned to each cross type. Fork length was the response variable in these models, and cumulative growing degree days (CGDDs) were included as continuous explanatory variables. Daily water temperature information was available from the Rough River over the duration of the study (2017–2021). Environmental data were collected from a fixed monitoring station 140 m upstream from the RRDT (Marine Institute, unpublished data). Water temperature was recorded every 30 min using a temperature sensor (HOBO TidbiT, Onset, MA, USA). CGDD was calculated using the formulation of McMaster and Wilhelm (1997) from an assumed egg fertilization date of January 1, 2017, as the sum of daily mean water temperatures that exceeded a lower thermal limit of 3.56°C and were less than an upper thermal limit of 19.48°C (Elliott et al., 1995). The experimental cross to which the fish assigned was included as a factor with six levels, and an interaction between CGDD and cross type was also included to test if growth rates of parr from each group differed as a function of temperature. The capture method (a two‐level factor: electrofishing or RRDT) was included to correct for any size differences between fish caught in the river versus fish leaving the river, and an interaction between CGDD and capture method was included to test for growth rate differences between electrofished versus trap‐caught fish. The response variable (fork length) was log_*e*_‐transformed prior to analysis to improve conformity with normality and homoscedasticity assumptions.

To test for differences in survival to spawning, fish sampled by electrofishing in the Rough River, fish captured at the RRDT, and fish detected on the array of antennas in the Rough River were classified as being putative spawners/non‐spawners using the methods described in Finlay et al. (2020). Briefly, if the fish was captured by hand and showed clear physical signs of maturation (males running milt, females with soft and bulging abdomens and a distended ovipositor) they were classed as putative spawners, whereas fish not showing such signs were classed as putative spawners if their projected size (based on a linear growth model) during a predefined spawning window of 1st of November to the 28th of February (across the years 2018–2021) was >165 mm.

All analyses were conducted in R version 4.3.1 (R Core Team, 2023) using the RStudio programming environment (Posit Team, 2023).

## 3 RESULTS

### 3.1 Representation in the river

**FIGURE 2:**
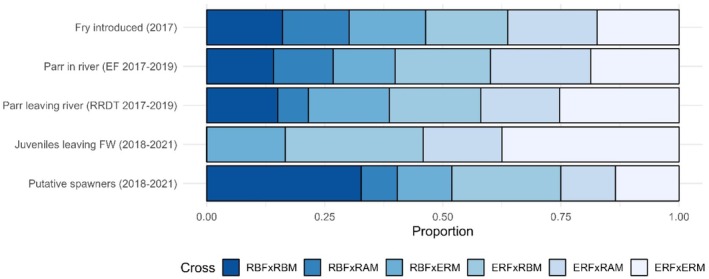
The proportions of *Salmo trutta* from the various samples assigned to each experimental cross type. EF, electrofishing. RRDT, Rough River Downstream Trap.

A total of 823 samples were obtained by electrofishing in the experimental river (the Rough) in 2017, of which 56 (6.8%) were assigned to experimental broodstock, that is, one of our six genetic crosses. After repeat samples were excluded on the same individual fish, a total of 49 individuals were there in the 2017 electrofishing sample. The proportions assigned to each cross type did not differ significantly from those expected based on the introduced fry (Table 1; Figure 2).

**TABLE 1:** Numbers of *S. trutta* assigned to each cross type for each sample.

Sample	RBF × RBM	RBF × RAM	RBF × ERM	ERF × RBM	ERF × RAM	ERF × ERM	χ^2^	*p*‐Value
Estimated fry introduced (aged 0+)	2057	1813	2078	2229	2429	2227		
EF17 (aged 0+)	7	4	9	7	15	14	6.816	0.245
EF18 (aged 1+)	23	23	20	35	41	30	6.03	0.303
EF19 (aged 2+)	4	5	5	7	8	3	2.472	0.79
Total parr in river (EF17 + EF18 + EF19)	34	32	34	49	64	47	8.249	0.141
RRDT17 (aged 0+)	6	1	14	17	10	14	14.781	**0.014**
RRDT18 (aged 1+)	17	7	17	16	16	23	6.139	0.294
RRDT19 (aged 2+)	5	4	4	8	8	10	3.319	0.654
Total parr leaving river (RRDT17 + RRDT18 + RRDT19)	28	12	35	41	34	47	15.952	**0.006**
Fish detected at lake entrance (Black River antenna)	8	2	9	10	8	8	4.113	0.557
Smolts + autumn parr leaving fresh water (sea‐entry traps)	0	0	4	7	4	9	14.813	**0.006**
Putative spawners	17	4	6	12	6	7	14.068	**0.019**

*Note*: The χ^2^ statistics and associated *p*‐values given in the last columns correspond to the results from a χ^2^ test comparing the observed proportions per cross type in that sample against the corresponding proportions in the introduced fry (row 1). Note that the EF17, EF18, and E19 rows do not sum exactly to the numbers given in the “Total parr in river (EF17 + EF18 + EF19)” row, because repeat captures on the same individuals were accounted for in the latter. Significant chi‐squared tests (*p*‐Value) are highlighted in bold.

Abbreviations: EF, electrofishing; RRDT, Rough River Downstream Trap.

In 2018 (aged 1+), a total of 1144 samples were obtained by electrofishing, with 172 (15.0%) assigned to experimental broodstock (representing 142 individuals). The proportions per cross type did not differ significantly from the introduced fry (Table 1; Figure 2). In 2019 (aged 2+), a total of 686 samples were obtained by electrofishing, with 32 (4.7%) assigned to experimental broodstock (representing 29 individuals). Again, the proportions per cross type did not differ significantly from the introduced fry (Table 1; Figure 2). Combining all 3 years, the proportions of parr (ages 0+, 1+, 2+) assigned to each cross did not differ significantly from the introduced fry (Table 1; Figure 2), despite the fact that the crosses were differentially represented at the RRDT (see Section 3.2). Electrofishing in 2020 yielded only 5 fish (out of a total of 568 sampled) that were assigned to one of our six crosses. The fork lengths of unassigned fish caught by electrofishing showed multimodal distributions each year, consistent with them representing a range of natal cohorts. Considering the assigned fish only, the sex ratio of aged 0+ parr electrofished in 2017 did not differ from parity (48.1% females; exact binomial test *p* = 0.513), but 1+ parr electrofished in 2018 were skewed toward males (43.4% females; *p* < 0.001), as were 2+ parr electrofished in 2019 (45.4% females; *p* = 0.027). Sample size did not allow testing for sex ratio biases within crosses.

### 3.2 Movements out of the Rough River

**FIGURE 3:**
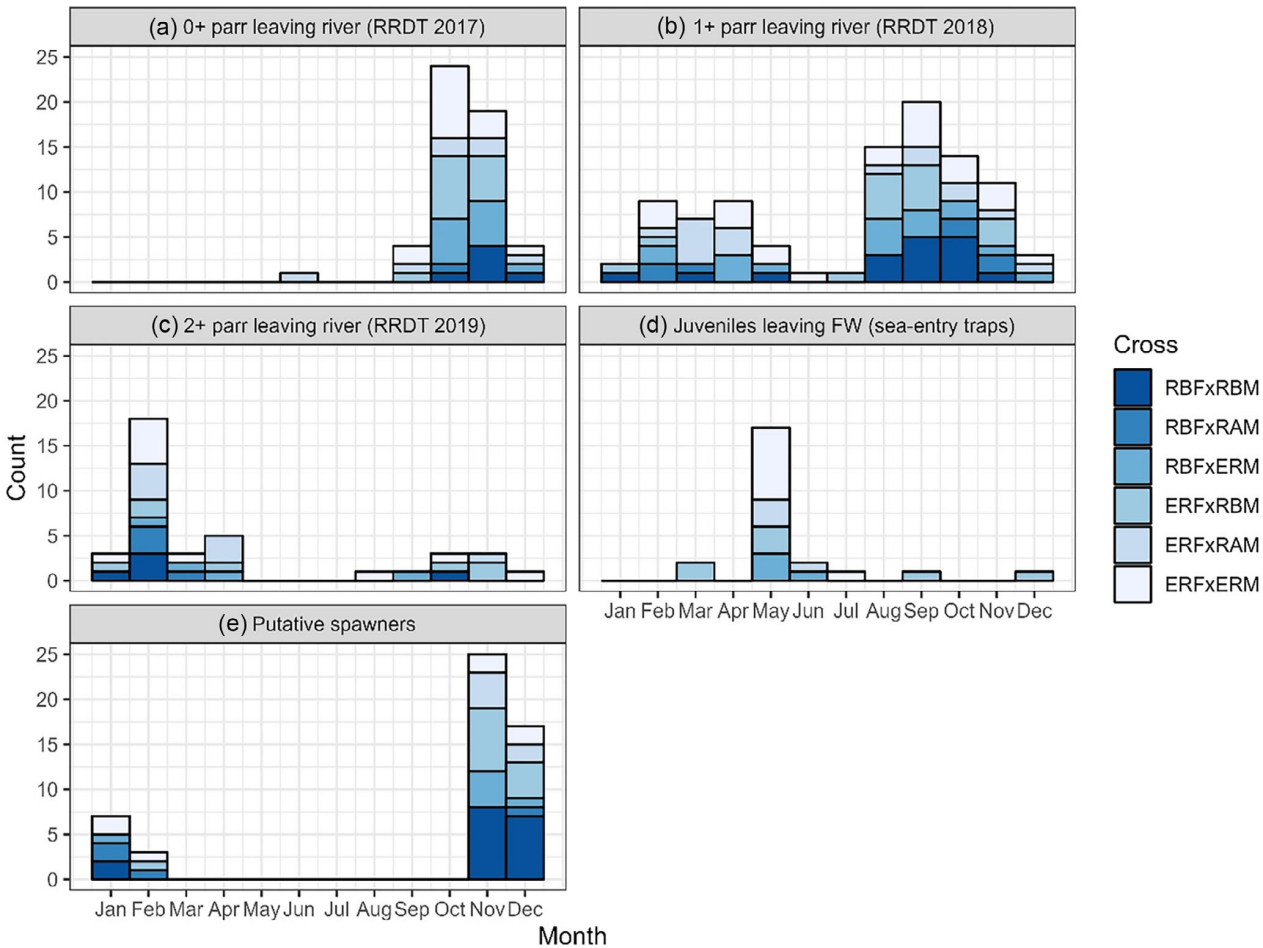
Histograms showing counts of *Salmo trutta* per month, broken down by experimental cross, for the various samples. RRDT, Rough River Downstream Trap.

Across the years 2017, 2018, and 2019, a total of 2245 *S. trutta* parr were sampled in the RRDT as they exited the Rough River, of which 198 (8.8%) were assigned to one of our six genetic crosses. The proportions per cross type differed significantly from those expected based on the introduced fry (χ^2^ = 15.42, *df* = 5, *p* = 0.014), which was largely driven by an excess of parr assigned to the ERF × ERM group and a deficit assigned to the RBF × RAM group (Table 1; Figure 2). Parr moved through the RRDT in distinct spring and autumn pulses (Figure 3a–c), particularly in 2018 (age 1+) and 2019 (age 2+). Of the 336 *S. trutta* sampled in the RRDT in 2020, only 0 (aged 3+) were assigned to our experimental broodstock, whereas of the 222 *S. trutta* sampled in the RRDT in 2021, only 1 (aged 4+) fish was assigned to the RBF × RBM cross. The numbers were too low for these 2 years to conduct meaningful tests for composition differences with respect to the introduced fry. The sex ratio of aged 0+ parr (assigned fish only) sampled in the RRDT in 2017 was skewed toward females (60.6%; exact binomial test *p* < 0.001), and the same was true for 1+ parr sampled in the RRDT in 2018 (60.4% females; *p* < 0.001). The sex ratio of 2+ parr sampled in the RRDT in 2019 did not differ from parity (52.7% females; *p* = 0.313).

### 3.3 Parr growth rates

**FIGURE 4:**
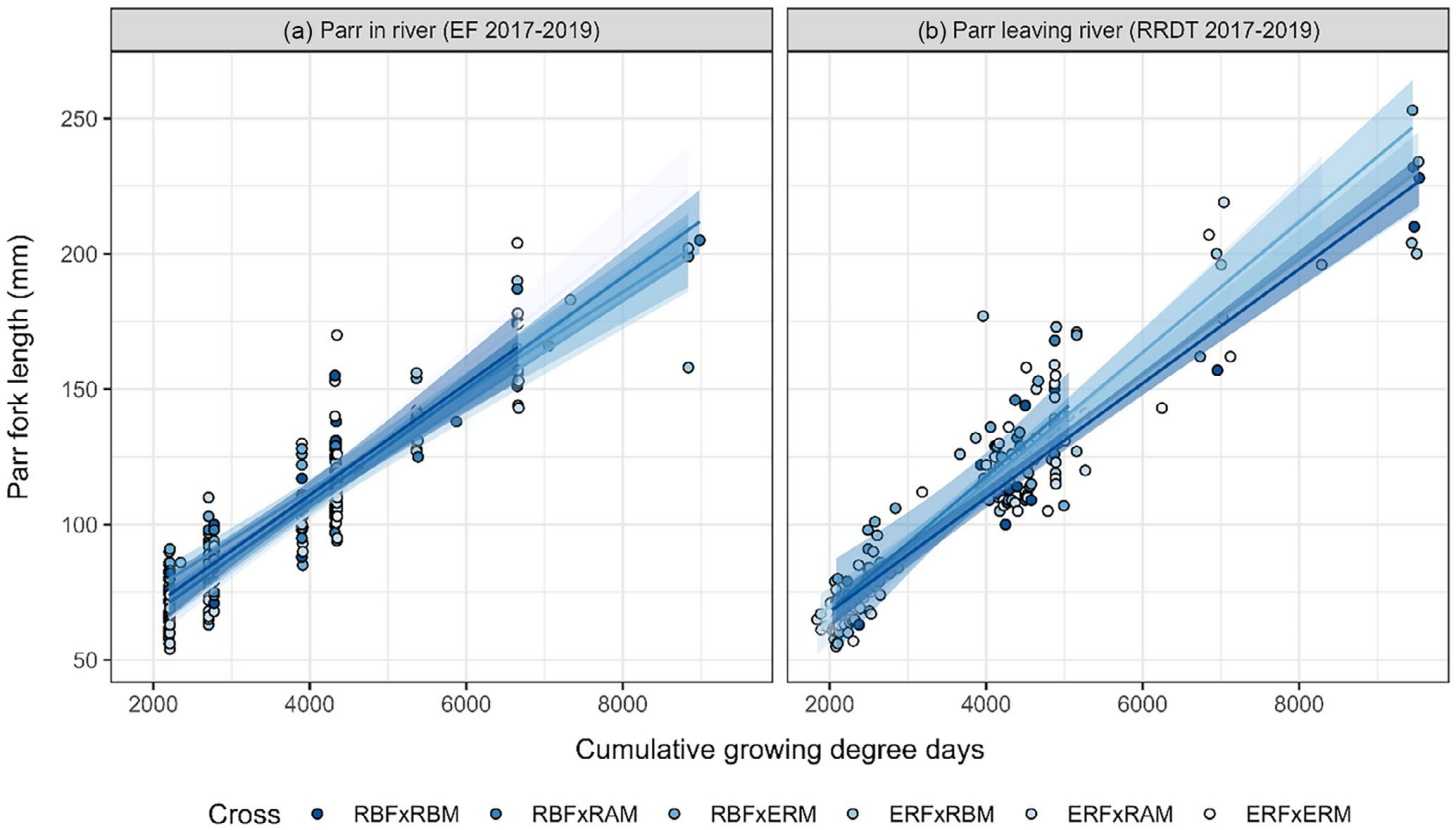
Fork length of parr sampled by electrofishing in (a) the Rough River or (b) the Rough River Downstream Trap (RRDT) between 2017 and 2019 as a function of cumulative growing degree days (CGDD). The regression lines and associated 95% confidence intervals are included for visual purposes only, as the underlying analysis was based on log‐transformed fish size. The relationships were approximately linear on the untransformed scale also, however.

When considering the years with higher sample sizes (2017–2019, ages 0+ to 2+), and pooling the electrofishing data with the RRDT data, significant differences (*F*_5,444_ = 2.96, *p* = 0.012) in mean parr size (log_*e*_ of fork‐length) were found in the growth model. Pair‐wise comparisons of each group against the RBF × RBM baseline showed that this overall effect was driven by the ERF × RAM group, with parr assigned to this group being on average 4.4% smaller than parr assigned to the RBF × RBM group (*p* = 0.028). The size differences among groups were therefore small (Figure 4), and none of the other groups differed significantly (all *p* > 0.05) from the baseline. The relationship between parr fork‐length and CGDD was linear on the natural log‐scale and approximately linear on the untransformed scale (Figure 4). The average growth rate was significantly higher (*F*_1,444_ = 9.42, *p* = 0.002) for fish sampled in the RRDT on exiting the Rough River, which grew by 0.023% for every unit increase in cumulative GDD, than fish sampled by electrofishing in the Rough River, which grew by 0.020% for each degree increase (Figure 4). There were no significant differences in growth rate among parr with respect to assigned cross type (*F*_5,439_ = 1.48, *p* = 0.193).

### 3.4 Smolts and autumn parr leaving fresh water

A total of 2182 *S. trutta* were sampled for genetics at the sea‐entry downstream traps between 2017 and 2021, of which 19 were assigned to the experiment. On top of these, an additional five PIT‐tagged fish belonging to the experiment (but not sampled again for genetics) were detected via the antennas/handheld readers at the sea‐entry traps, giving *n* = 24 fish in total assigned to the experiment. Their group composition differed significantly from the group composition of the introduced fry (Table 1; Figure 2) and from the group composition of the parr sampled in the Rough River (EF 2017–2019; χ^2^ = 12.27, *df* = 5, *p* = 0.027). Importantly, all of these 24 fish were assigned to crosses involving one or two Erriff parents, and none were assigned to the RBF × RAM or RBF × RBM cross types. There was an excess of offspring assigned to the ERF × EFM group (37.5%) and the ERF × RBM (29.2%) compared to the introduced fry (17.3% and 17.4%, respectively). Two of the 24 fish outmigrated as autumn parr in September 2018 (aged 1+) and December 2019 (aged 2+), and a third fish was also brown in colouration but outmigrated in March 2019 (aged 2+). The other 21 fish outmigrated as smolts in the spring/early summer (Figure 3e), with 11 of them outmigrating in 2019 (aged 2+), 7 of them outmigrating in 2020 (aged 3+), and 3 outmigrating in 2021 (aged 4+). The *n* = 24 downstream migrants detected at the sea‐entry traps were skewed toward females (66.7%), but the difference was not statistically significant (χ^2^ = 2.00, *df* = 1, *p* = 0.157).

### 3.5 Putative spawners

A total 52 assigned fish (24 females, 27 males, and 1 individual of unknown sex) were deemed to be putative spawners. Only one of these (assigned to the ERF × RBM group) was detected at the sea‐entry traps also, on March 12, 2019 (aged 2+), but this was 27 days after the fish had been detected as a putative spawner in the RRDT (on February 13, 2019), suggesting that it spawned in the Rough River and then migrated immediately to the sea. Four of the 52 putative spawners were deemed to have spawned in 2018 (at age 1+; 3 males and 1 female), 20 in 2019 (age 2+; 14 males and 6 females), 12 in 2020 (age 3+; 7 males and 5 females), and 16 in 2021 (age 4+; 3 males, 12 females, and 1 unknown sex).

The proportions of the putative spawners assigned to each cross type differed significantly from the corresponding proportions in the introduced fry (Table 1; Figure 2). They also differed significantly (χ^2^ = 15.31, *df* = 5, *p* = 0.009) from the corresponding proportions in the parr sampled by electrofishing in the Rough River between 2017 and 2019, and from the corresponding proportions in the juvenile outmigrants sampled at the sea‐entry traps (χ^2^ = 15.10, *df* = 5, *p* = 0.010). In particular, there was an excess of putative spawners assigned to the RBF × RBM group (32.7%) relative to the introduced fry (16.0%), the electrofished parr 2017–2019 (13.1%), and the seaward migrants (0%), suggesting that pure Rough River fish were more likely than the other groups to survive to spawning.

## 4 DISCUSSION

The results of our CGE, conducted under wild conditions, generally supported our hypothesis that anadromous migration tendency depends on genetic origin. Seaward migrating juveniles sampled at the sea‐entry traps were assigned disproportionately to crosses involving one or two Erriff parents, with a deficit assigned to crosses involving two Burrishoole parents. As predicted a priori, the highest smoltification rate was observed in the ERF × ERM cross, with 37.5% of the smolts assigned to this group (compared to an initial estimated representation of 17.3% in the introduced fry), and the lowest smoltification rates were observed in the RBF × RAM and RBF × RBM groups (no smolts were assigned to either cross, despite them representing an estimated 14.1% and 16.0%, respectively, of the introduced fry). Although the overall number of smolts produced by the experiment was low (*n* = 24 out of ~12,800 initial fry), this was still a large enough sample to be able to detect statistically significant differences with respect to cross type. Unfortunately, it was not possible to include a pure RAF × RAM group in the experimental design, but the offspring from such a cross would be expected to have an even lower smolt migration tendency than any of our six groups, given that selection in the above‐falls section of the Rough River should disfavor anadromy (Moran et al., 2024; Phillis et al., 2016) and favor the ability of juveniles to hold position in the stream (Jonsson, 1982).

Local adaptation, which has been documented before in brown trout at fine spatial scales (Westley et al., 2013; but see Stelkens et al., 2012; Labonne et al., 2021) and in Atlantic salmon (*Salmo salar* L.) in a previous CGE undertaken in the Burrishoole catchment (O'Toole et al., 2015), was an important factor to consider in our study design. If the offspring of Burrishoole (RB or RA) parents were more likely to survive to smolt age because of a “home advantage” than the offspring of Erriff parents, then smolt numbers would be skewed toward the former even if there were no inherited differences in anadromy tendency. We found a skew in the opposite direction, however. Although we did not observe any obvious differences in early survival, as measured by the representation of the various crosses in the Rough River at the parr stage (electrofishing data), we did find that offspring from the RBF × RBM and RBF × RAM crosses were more likely to become spawners than those from crosses involving one or two Erriff parents, implying performance differences across the life cycle. None of our putative spawners had previously been sampled as a smolt at the sea‐entry traps, implying that any group‐specific survival differences likely occurred in fresh water rather than at sea. For example, fish with Erriff alleles that moved downstream out of the Rough River may have been less equipped to survive a period of feeding or passage through Lough Feeagh, given that the Erriff system has much less lacustrine habitat. Thus, if anything, inherent differences among these populations in the propensity for anadromy may be even stronger than suggested by our smolt numbers. It is also notable that we found effects of genetic origin on the propensity of fish to move downstream as parr out of the Rough River (RRDT data), which were broadly consistent with the patterns observed in the smolts.

Previous work on brown trout from the Burrishoole and Erriff catchments (Archer et al., 2019, 2020) has explored similar questions, utilizing laboratory experiments undertaken in an indoor recirculating aquaculture system. Such experiments have the advantage that environmental conditions (e.g., temperature, water chemistry, food) and other parameters such as fish density and composition can be tightly controlled or manipulated, but the downside is that results might not translate to wild environments, especially if traits of interest are subject to strong genotype‐by‐environment (G × E) interactions (Hutchings, 2011). Archer et al. (2019) found that a higher fraction of purebred Erriff offspring underwent a parr‐to‐smolt transformation in the tank environment compared to the purebred offspring of broodstock obtained from Lough Bunaveela (Figure 1), a putative lake‐resident population in the Burrishoole system. Fish from the two population backgrounds also responded differently to food deprivation manipulations, implying G × E interactions. Interestingly, some of the Bunaveela fish became smolts under low food treatments, albeit at a lower rate than the Erriff fish (Archer et al., 2019), implying that migration capacity can lie dormant in populations that are currently non‐migratory in the wild, consistent with threshold models of migration (Buoro et al., 2012; Buzatto et al., 2015). Our current results are also broadly consistent with those of Vainikka et al. (2023), who conducted a CGE with brown trout in outdoor circular channels—a “middle ground” between indoor tanks and a fully wild environment—and found intrinsic differences in migration tendency among purebred, crossbred, and backcrossed groups of migratory, resident, and hybrid strains of *S. trutta* originating from a single catchment in north‐central Finland. Vainikka et al. (2023) assessed individual migratory behavior in a continuous manner, rather than dichotomously as we (and Archer et al.) did, and noted that it is unclear how migration distances measured in experimental circular channels relate to visual indicators of smoltification and the actual tendency to perform lakeward or seaward migration. Individual migration distance in their study was nevertheless strongly positively correlated with physiological smoltification (gill Na+/K+ ATPase concentration and activity), and the latter also differed significantly with respect to genetic origin (see also Archer et al., 2019), being higher in the migratory strain even though the natural destination is a lake, rather than the sea. Collectively, the findings of these CGEs, as well as the field transplant experiment of Jonsson (1982), point toward genetic differences among populations in suites of traits comprising the migratory syndrome (c.f. Dingle & Drake, 2007).

In recent years there has been great interest in understanding the genomic architecture of migratory life histories in salmonids (Hale et al., 2013; Hecht et al., 2012; Kelson et al., 2019; Kjærner‐Semb et al., 2021; Lemopoulos et al., 2018, 2019; Micheletti et al., 2018; Moran et al., 2024; Pearse et al., 2019; Prince et al., 2017; Salisbury et al., 2022; Thompson et al., 2019). The genetic architecture underlying alternative migratory tactics in brown trout remains largely unknown, but it seems to be highly polygenic, with many candidate genes involved (Lemopoulos et al., 2018, 2019; Moran et al., 2024). In rainbow/steelhead trout (*Oncorhynchus mykiss*), migratory versus resident life history is associated with polymorphism at a 55‐Mb double‐inversion supergene (Arostegui et al., 2019; Pearse et al., 2019). Suites of genes have been found to be differentially expressed between smolts and residents in *S. trutta* (Giger et al., 2006; Wynne et al., 2021) and *Oncorhynchus mykiss* (Hale et al., 2016), or to be differentially methylated (Baerwald et al., 2016), but these may reflect downstream consequences of an initial decision made early in ontogeny under the control of different genes, for example, master regulatory switches (Ferguson et al., 2019). Future studies should thus strive to identify genetic polymorphisms underpinning variation among individuals or populations in the migration threshold or underlying liability trait(s), and the subsequent chain of gene expression changes (likely under epigenetic regulation) through which different genotypes develop along different developmental trajectories (Aubin‐Horth et al., 2005; Aubin‐Horth & Renn, 2009; Dodson et al., 2013). We also note that although a continuum of life‐history tactics may occur at the phenotypic level (Birnie‐Gauvin et al., 2021; Cucherousset et al., 2005), this diversity may nevertheless stem from a series of sequential binary decisions at different time points in ontogeny (Thorpe et al., 1998).

Poole et al. (2007) speculated that the sudden and sustained drop in marine survival rates that occurred in the Burrishoole catchment from the late 1980s onwards may have led to an evolutionary response toward reduced anadromy. This would require the propensity for marine migration to be under strong genetic control, which is supported by the fact that smolt production is tightly related to the level of ova deposited by anadromous returns (de Eyto et al., 2016; Poole et al., 2007). Alternatively, the collapse in the anadromous component of the stock could reflect a phenotypically plastic response to changing freshwater conditions. Although the Burrishoole catchment has experienced considerable land use change in recent decades, this does not appear to have impacted the freshwater survival of trout or salmon in the system (de Eyto et al., 2016). Improved growth in fresh water owing to a warming climate (de Eyto et al., 2022) may have reduced the need for marine migration, but the steady warming that has occurred since the 1970s may not be sufficient to explain the sudden sea trout collapse. Although smolt output decreased, egg‐to‐smolt survival increased after 1989, suggestive of relaxed density dependence associated with the substantial drop in egg deposition by marine‐returning fish. Improved per‐capita freshwater growth would then further reduce the need for a marine phase to the life cycle. In reality, the stark changes in population dynamics may reflect some combination of plastic responses to changing freshwater conditions and an evolutionary response to reduced marine survival as a result of sea lice parasitism (Gargan et al., 2017; Thorstad et al., 2015). Although the exact spawning locations of anadromous fish in the Burrishoole remain unclear, it has long been thought that the Rough River was a major contributor to past sea trout runs (J. Magee, unpublished PhD thesis). In our current study, the RB × RB cross produced no smolts: this result is consistent with the genetic tendency for anadromy being very low nowadays in the Rough River below‐falls population, and we speculate that this tendency to migrate was likely considerably higher prior to the sea trout collapse. Rapid evolution in migratory tactics has been observed in *O. mykiss* (Phillis et al., 2016), and modeling studies suggest that it can occur in management‐relevant timeframes in response to anthropogenic pressures in the marine environment (Kane et al., 2022; Theriault et al., 2008). It could also be that distinct anadromous subpopulations within the Burrishoole catchment were lost during the collapse, but further population genetic work comparing contemporary and historic samples is required to test this.

The Erriff stock also underwent substantial changes in life‐history composition shortly after the establishment of commercial Atlantic salmon farms in the local estuary, with reductions in the size and number of sea trout kelts, the sea trout rod catch, the estimated numbers of eggs deposited, the proportion of older (1+ and 2+ sea age) fish, and the frequency of repeat spawners (Gargan et al., 2016). The kelt numbers had recovered substantially by the late 1990s/early 2000s; however, the anadromous run in the Burrishoole has remained very low. It may therefore be that sea‐lice‐related marine selection pressures have been weaker or more variable in the Erriff system, which has thus retained a higher genetic propensity for anadromy than the Burrishoole. The systems differ in other notable respects too, however. For example, the brackish lagoon (Lough Furnace) in the Burrishoole system may provide habitat options for semi‐anadromous trout that leave freshwater “proper” but do not enter full seawater (Birnie‐Gauvin et al., 2019; Wynne et al., 2023). The much greater extent of freshwater lentic habitat in the Burrishoole compared to the Erriff may also favor potamodromous migration (Ferguson et al., 2019; Nevoux et al., 2019), which in turn may facilitate the evolutionary maintenance of migratory alleles per se. This may explain why a small smolt run still occurs in the system, and also why proportionally more offspring from the RB × RB cross moved downstream out of the Rough River than did offspring from the RB × RA cross (which are carrying putative resident alleles).

As with all logistically challenging field experiments with long‐lived organisms, our study had some limitations. First, it was conducted only in the Burrishoole environment: a reciprocal transplant experiment would have been required to disentangle genetic from environmental differences between populations in migration tendencies. For example, smoltification rates for all crosses may have been higher if the offspring were reared in the Erriff environment should resources be more limiting there (Archer et al., 2019, 2020; Olsson et al., 2006; Peiman et al., 2017; Wysujack et al., 2009). Second, ideally CGEs or reciprocal transplant experiments should have replication at the population level, for example, multiple migratory strains and multiple resident strains, but in reality this is very challenging. Third, we could not exclude the possibility that observed differences with respect to cross type might reflect nongenetic parental effects (Hughes et al., 2018; Van Leeuwen et al., 2017), such as maternal effects or epigenetic inheritance (Wellband et al., 2021), but genomic analyses on Irish brown trout populations do suggest adaptive divergence at a DNA sequence level with respect to migratory life histories (Moran et al., 2024). Fourth, we did not know the actual migratory phenotypes of the parents in our crosses, so our inferences were limited to the population level, rather than the individual or family level. It is noteworthy, though, that differences in migration tendencies were still found, even though other catchments in the region (e.g., the Owenduff River, Co. Mayo) might have an even‐higher genetic propensity to produce sea trout than the Erriff. Finally, we did not have an exact count of the number of unfed fry stocked out, rather just an estimate, but the fact that no significant differences were found between these estimates and the numbers of 0+ parr sampled in the river by electrofishing (Table 1) suggests that the estimates were accurate, assuming no intervening group‐specific differences in fry survival.

In conclusion, we have presented a CGE undertaken under wild conditions that are consistent with heritable life‐history differences among geographically proximate brown trout populations that may also be locally adapted to the unique conditions of their home environments. As typically found in salmonids (Ferguson et al., 2019), migration rates were higher in females than in males in our study, presumably underpinned by sex‐specific genetic architecture (c.f. Barson et al., 2015; Pearse et al., 2019), a possibility that warrants further study in brown trout. A genetic basis to migration means that stocks are not readily interchangeable in a transplanting/stocking context. That said, ecological restoration programmes may, in certain situations, benefit by choosing a genetically diverse source population that exhibits a mix of migrant and resident phenotypes, such as to facilitate the colonization of upper reaches of the catchment that require residency or potamodromy. Brown trout have been stocked all over the world outside of their native Eurasian range, and the capacity for facultative anadromy as well as rapid evolution likely played a strong role in successful early establishments (Davidsen et al., 2021; Labonne et al., 2013; O'Toole et al., 2021; Westley et al., 2013). Conserving genetic diversity within and among populations and the associated continuum of life histories will foster increased resilience in the face of climate change, particularly for stocks subject to additional pressures such as intensive exploitation, sea lice from fish farms, or detrimental stocking practices.

## AUTHOR CONTRIBUTIONS

Conceptualization: Thomas E. Reed, Philip McGinnity, Russell Poole, Patrick Gargan, and Robert Wynne. Developing methods: all authors. Data analysis: Thomas E. Reed, Robert Wynne, Jamie Coughlan, Joshka Kaufmann, Karl. P. Phillips, and Adrian Rinaldo. Preparation of figures and tables: Thomas E. Reed and Robert Wynne. Conducting the research, data interpretation, writing: all authors.

## FUNDING INFORMATION

TER and RW were supported by an ERC Starting Grant (639192) and an SFI ERC Support Award. JK, KPP, and PMcG were supported by Science Foundation Ireland, the Marine Institute Ireland and the Department for the Economy, Northern Ireland, under the Investigators Program grant number SFI/15/IA/3028. PMcG was further supported by the Marine Research Programme Awards RESPI/FS/16/10 and RESPI/BIO/21/01. JK is currently supported by Taighde Éireann—Research Ireland, under the Pathway Programme (grant no.: SFI‐IRC/21/PATH‐S/9671). RP and data collection were supported by the Marine Institute, Ireland. AR was supported the Marie Skłodowska‐Curie Programme, grant no. 956623, MSCA‐ITN‐ETN‐European Training Network, inventWater (https://inventwater.eu/).

## Supporting information


**Data S1.** Supporting information.
