# Supplementary material for: A common garden experiment in the wild reveals heritable differences in migration tendencies among brown trout populations
Source: J Fish Biol. 2025 Feb 6;106(6):1816–30. doi: 10.1111/jfb.16068 (PMC12244308; doi:10.1111/jfb.16068)
Supplement: Supplementary file 1 — Data S1. Supporting information. [file JFB-106-1816-s001.docx]

**Supplementary materials for Reed et al.: “Heritable differences in migration tendencies among geographically proximate brown trout populations revealed by a common garden experiment in the wild.”**

1. **Genetic differences among the three source populations**

To examine neutral genetic divergence among the three source populations, genotypic information was used from 16 microsatellite loci (see section 3 below) for the male broodstock used in the experiment (Rough Above-Falls n=26; Rough Below-Falls: n=64; Erriff: n= 57). Males only were considered here because we only had male broodstock for the Rough Above-Falls population, so females from the Rough Below-Falls and Erriff populations were excluded to avoid biases (but the results were very similar if these females were included in the analysis). Blind clustering using the *find.clusters()* function in the R package *adegenet* (Jombart et al. 2008) showed that the most likely number of clusters (lowest BIC) was three, corresponding to the actual number of putative populations. A discriminant analysis of principal components (DAPC) using the *dapc()* function (Jombart et al. 2010) showed reasonably strong separation among the three populations, with the Rough-Above Falls (RA) population being the most different (Fig. S1).


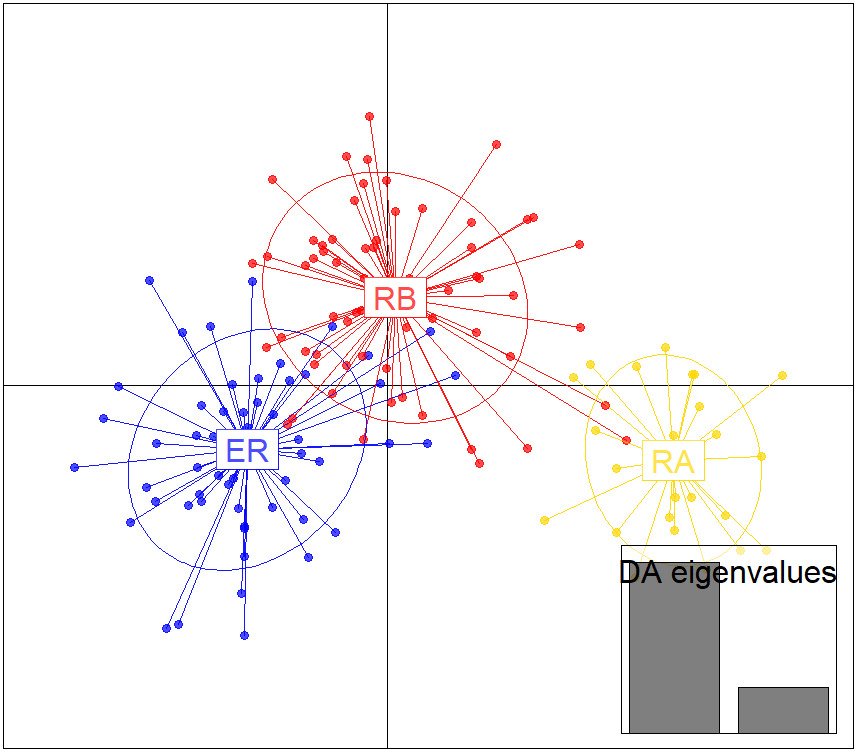


Fig S1: Distinct genetic profiles between Erriff (ER), Rough Above-Falls (RA) and Rough Below-Falls (RB) male broodstock based on 16 microsatellite loci. Scatterplot displaying the results of a DAPC based on 3 retained principal components (the optimal number as indicated by the *optim.a.score* function).

Exact G tests for genic differentiation among the populations were run in Genepop 4.7.5 (Raymond & Rousset 1995; Rousset 2008) using default parameters, which revealed highly significant differences across all loci at the global level and for each population pair (all P<0.001).

1. **Broodstock, crossing procedures and hatchery rearing.**

Broodstock for the experiment were caught from the Rough Below-Falls and Rough Above-Falls populations by electrofishing in Nov/Dec 2016, and from the Erriff system in Nov/Dec 2016 by electrofishing and seine-netting at various sites along the system. This sampling was broadly representative in all three cases of the range of adult sizes and phenotypes present in each population but note that the migration history of each individual fish was unknown. The goal here was not to target either anadromous or resident fish specifically, but rather to representatively sample each population.

A total of 95 adult female brown trout (n=41 Erriff females; n=54 Rough Below-Falls females) and 147 adult male brown trout (n=57 Erriff males; n=26 Rough Above-Falls males; n=64 Rough Below-Falls males) were captured from the wild to form potential broodstock for the experiment and held in holding tanks until stripping. Gamete stripping was conducted across four dates (Dec 6^th^, 14^th^, 15^th^ and 21^st^ 2016), with approximately equal numbers of males and females from each population stripped on each day (with some males stripped on multiple days). On the day of stripping, all fish were weighed, measured and photographed, and then stripped of their gametes. A volumetric egg count was recorded for each female. Not all captured males were stripped; of the 147 individuals captured, 121 ended up producing milt.

For 91 of the females, their eggs were split into three approximately equal sized batches and each batch was fertilised by the milt of a different male (one Erriff, one Rough Below-Falls, and one Rough Above-Falls sire). For the other four females (two Erriff and two Rough Below-Falls), their eggs were split into only two batches, with each fertilised by two males (one Erriff and one Rough-Below). Initially there were 18,590 fertilized eggs across 281 full sibling families in total. Batches within females were mixed post-fertilisation and the eggs were then incubated in separate flume sections according to mother (i.e. maternal half-sibling families) at an experimental hatchery facility operated by the Marine Institute Newport, in the Burrishoole catchment, supplied with water piped directly from Lough Feeagh. Egg mortalities were counted daily per section during this initial incubation phase.

After around 5 weeks (24^th^ to 26^th^ of January 2017), all eggs were physically shocked by mechanically agitating the eggs, so that the infertile and dead eggs turned white and could be easily separated from the fertile healthy ones, counted, and removed. Post-shocking, there were 15,506 viable eggs remaining, comprising 84 of the original 95 maternal half-sibling families (Erriff females: 8831 eggs; Rough Below-Falls females: 6675 eggs). At this stage the eggs were randomly mixed (i.e., no longer kept separately based on mother) and transferred to a new set of trays, in which they were incubated through until hatching. The unfed fry were then stocked out into the Rough River (randomly distributed through the experimental stretch of stream) in two separate batches on the 10^th^ March 2017 (n=6494 fry) and 23^rd^ March 2017 (n=6339 fry). Because eggs from different sires within mothers were mixed prior to egg-shocking, and eggs from different mothers were mixed post-shocking, accurate counts of the number of unfed fry stocked out per full-sibling family were not available. Estimates were instead obtained by assuming that egg survival was random with respect to cross type (see Table 1 in main text). Whilst families may have differed in their realised egg survival, any such differences are likely to have averaged out with respect to cross type. Genomic incompatibilities in hybrid crosses, for example, would only show up in the second (F2) or later generations, whereas our design only entailed F1 hybrids.

1. **Molecular work**

Genomic DNA was extracted using the Promega Wizard® SV 96 Genomic DNA Purification System. DNA quality and quantity were assessed on agarose gels by comparison with a Quick-Load® Purple 100 bp DNA Ladder (New England Biolabs). Concentration of DNA for PCR was adjusted to 2–10 ng μl−1.

Multiplex PCRs were used to amplify 17 loci (16 microsatellite loci and the SalmoY sex identity locus) in two separate reactions (panels). Forward primers were labelled with fluorescent labels from the Applied Biosystems (ABI) standard dye sets to enable visualisation on ABI genetic analysers, and reverse primers included a GTTT “pig tail” to minimise stuttering. In panel 1, a single set of primers (One102) amplified two distinct and apparently unlinked loci which could be scored effectively. Primer sequences were taken from relevant publications (as cited in Wynne et al. 2023) for the following loci: Ssa197, Ssa85, SsaD71, Ssa410, Ssa416, CAO48828, CAO53293, CAO60177, One102a and One 102b (primer sequences for this locus amplify two unlinked microsatellite loci), One103, One108, ppStr3, Cocl-lav-4, SasaTAP2 and One9Asc. All PCRs were performed in a total volume of 3.5 μl, including 2–10 ng of genomic DNA and 1.75 μl Plain Combi PPP Master Mix (TopBio). Primer concentrations (same for forward and reverse primers for each locus) and fluorescent label employed in each panel were as follows:

***Panel 1.*** *SalmoYF(VIC) – 0.03 μM, Ssa85(NED) – 0.04 μM, Oneu9ASC (VIC) – 0.05 μM, Ssa416UOS (FAM) – 0.06 μM, One102 (NED) – 0.08 μM, CAO48828 (VIC) – 0.08 μM, One103 (FAM) – 0.1 μM, CAO53293 (PET) – 0.15 μM, CoclLav4 (VIC) – 0.2 μM, One108 (VIC) – 0.25 μM*.

***Panel 2.*** *Ssa197 (VIC) – 0.04 μM, SsaD71 (NED) – 0.05 μM, ppStr3 (FAM) – 0.06 μM, SasaTAP2 (NED) – 0.08 μM, CAO60177 (FAM) – 0.2 μM, Ssa410UOS (PET) – 0.25* *μM*.

Cycling conditions were as follows: Initial denaturation at 95°C for 15 min, followed by 25 cycles of 94°C for 30 s, 55°C for 60 s and 72°C for 90 s. A final extension step of 30 min at 60°C was performed. Electrophoresis was performed on an ABI3500xl DNA analyser (Applied Biosystems) using POP-7™ Polymer. Each sample was prepared in Hi-Di™ Formamide with GeneScan™ 600 LIZ™ Dye Size Standard (ThermoFisher Scientific) as a size ladder with which to compare allele size. Allele calling was conducted in GeneMarker (SoftGenetics).

**References:**

Jombart T. (2008) adegenet: a R package for the multivariate analysis of genetic markers Bioinformatics 24: 1403-1405. doi: 10.1093/bioinformatics/btn129

Jombart T, Devillard S and Balloux F (2010) Discriminant analysis of principal components: a new method for the analysis of genetically structured populations. BMC Genetics 11:94. doi:10.1186/1471-2156-11-94

Raymond M. & Rousset F, 1995. GENEPOP (version 1.2): population genetics software for exact tests and ecumenicism. J. Heredity, 86:248-249

Rousset, F., 2008. Genepop'007: a complete reimplementation of the Genepop software for Windows and Linux. Mol. Ecol. Resources 8: 103-106.

Wynne, R., Kaufmann, J., Coughlan, J., Phillips, Karl. P., Waters, C., Finlay, R. W., … Reed, T. E. (2023). Autumn outmigrants in brown trout (*Salmo trutta*) are not a demographic dead-end. *Journal of Fish Biology*, *102*, 1327–1339.
